# A Novel Mutation in *COL4A1* Gene in a Chinese Family with Pontine Autosomal Dominant Microangiopathy and Leukoencephalopathy

**DOI:** 10.1007/s12975-021-00926-0

**Published:** 2021-08-20

**Authors:** Qing Li, Chengfeng Wang, Wei Li, Zaiqiang Zhang, Shanshan Wang, Autongsha Wupuer, Xiao Hu, Kalibinuer Wumaier, Yi Zhu, Hongyan Li, Wengui Yu

**Affiliations:** 1 Department of Neurology, People’s Hospital of Xinjiang Uygur Autonomous Region, Tianchi Road No. 91, Ürümqi, 830000 China; 2 Department of Neurology, University of California Irvine, Irvine, CA USA; 3 Department of Neurology, Beijing Tiantan Hospital, Capital Medical University, Beijing, China; 4 Monogenic Disease Diagnosis Center for Neurological Disorders, Beijing Tiantan Hospital, Capital Medical University, Beijing, China; 5 Precision Medicine Research Center for Neurological Disorders, Beijing Tiantan Hospital, Capital Medical University, Beijing, China; 6 China National Clinical Research Center for Neurological Diseases, Beijing Tiantan Hospital, Capital Medical University, Beijing, China; 7 Orange, CA USA

**Keywords:** Stroke, Hereditary, Cerebral small vessel disease, *COL4A1*

## Abstract

Pontine autosomal dominant microangiopathy and leukoencephalopathy (PADMAL) is a rare hereditary cerebral small vessel disease. We report a novel collagen type IV alpha 1 (*COL4A1*) gene mutation in a Chinese family with PADMAL. The index case was followed up for 6 years. Neuroimaging, whole-exome sequencing, skin biopsy, and pedigree analysis were performed. She initially presented with minor head injury at age 38. MRI brain showed chronic lacunar infarcts in the pons, left thalamus, and right centrum semiovale. Extensive workup was unremarkable except for a patent foramen ovale (PFO). Despite anticoagulation, PFO closure, and antiplatelet therapy, the patient had recurrent lacunar infarcts in the pons and deep white matter, as well as subcortical microhemorrhages. Whole-exome sequencing demonstrated a novel c.*34G > T mutation in the 3′ untranslated region of *COL4A1* gene. Skin biopsy subsequently demonstrated thickening of vascular basement membrane, proliferation of endothelial cells, and stenosis of vascular lumen. Three additional family members had gene testing and 2 of them were found to have the same heterozygous mutation. Of the 18 individuals in the pedigree of 3 generations, 12 had clinical and MRI evidence of PADMAL. The mechanisms of both ischemic and hemorrhagic stroke are likely the overexpression of *COLT4A1* in the basement membrane and frugality of the vessel walls. Our findings suggest that the novel c.*34G > T mutation appears to have the same functional consequences as the previously reported *COL4A1* gene mutations in patients with PADMAL and multi-infarct dementia of Swedish type.

## Introduction

Cerebral small vessel disease (cSVD) is a main cause of stroke, cognitive impairment, and vascular dementia [1]. Hereditary cSVD is rare, and mainly occurs in young adults [2, 3]. Cerebral autosomal dominant arteriopathy with subcortical infarcts and leukoencephalopathy (CADASIL), caused by *NOTCH3 gene* mutation [4], is the most prevalent monogenic cSVD [5, 6]. *NOTCH3 gene* encodes the *NOTCH3* receptor protein that is expressed predominantly in vascular smooth muscle cells to maintain vascular contractility. Despite widespread deposits of mutant *NOTCH3* protein in small arteries and capillaries, the clinical manifestations of CADASIL are only related to the central nerve system, in the form of migraine, acute encephalopathy, lacunar infarcts, cognitive impairment, gait, and mood disturbances [5–7].

Type IV collagen is a basement membrane protein expressed in blood vessels and organs throughout the body. Gene mutations in collagen type IV alpha 1 and alpha 2 chain (*COL4A1/2*) on chromosome 13q34 have been linked to a spectrum of cSVD in human, including perinatal intracerebral hemorrhage with consequent porencephaly, adult-onset intracerebral hemorrhage, lacunar infarcts, and leukoaraiosis [8–12]. Most *COL4A1/2* mutations are missense mutations involving a glycine residue in the translated regions, leading to the synthesis of an abnormal protein and vessel wall fragility [10–12].

Recently, mutations causing overproduction of *COL4A1* have been identified in patients with pontine autosomal dominant microangiopathy with leukoencephalopathy (PADMAL) [13] and multi-infarct dementia of Swedish type [14]. Three different heterozygous mutations, including c.*32G > T, c.*32G > T, and c.*35C > A, in the 3′ untranslated region of *COL4A1*, were identified in patients with PADMAL [13] while a c.*32G > A mutation was found in patients with multi-infarct dementia of Swedish type [14]. These variants affect a binding site of miR-29 microRNA and lead to upregulated expression of *COL4A1* [13, 14]. The clinical features of both diseases include leukoencephalopathy and multiple lacunar infarcts with pontine involvements [13–17]. Autopsy study showed massive fibro-hyalinosis and elastosis in small arteries and arterioles with atrophy of the tunica media and concentric intimal proliferation [13]. Strong staining with collagen IV–specific antibodies demonstrated fibrosis of the subendothelial space and the lamina muscularis. Electron microscopy (EM) analysis showed thickening of the basal lamina in cerebral vessels. Skin biopsy showed similar abnormalities in the basement membrane and vessel walls [13, 18].

In a recent case report of a Chinese PADMAL pedigree, MRI brain showed multiple lacunar infarcts in the deep white matter, pons, and cervical spinal cord [18]. Genetic analysis showed a c.*32G > A mutation in the 3′ untranslated region of *COL4A1*, which was identical to the mutation described in patients with multi-infarct dementia of Swedish type [14]. Another case report described a novel c.*33 T > A mutation in a patient with progressive gait disturbance and cognitive impairment [19]. MRI showed severe white matter disease; multiple lacunar infarcts with pontine involvement; and multiple microbleeds in the subcortical regions, brain stem, and cerebellum. In addition, there were superficial cortical siderosis at the right temporal lobe and hemorrhagic changes at the splenium of the left corpus callosum. Overexpression of *COL4A1* appears to interrupt the integrity of the basement membrane and cause both ischemic and hemorrhagic stroke [19]. These recent case reports suggest that PADMAL and multi-infarct dementia of Swedish type likely belong to a growing spectrum of the same hereditary cSVD.

Here, we report a novel c.*34G > T mutation in a Chinese PADMAL pedigree.

## Methods

### Index Case

The index case (III10) was a 38-year-old woman who had a minor head trauma in 2014. Workup with a brain magnetic resonance image (MRI) showed cSVD. She was therefore referred to our center for further evaluation. Over the next few years, she had recurrent ischemic stroke in the pons, thalamus, and subcortical white matter of unknown etiology. Extensive investigations included blood test, neuroimaging, cardiac workup, and gene testing for common monogenic cSVD.

### Genetic Analysis

Whole-exome sequencing was performed for the index case (III10) by the Running Gene Inc. (Beijing, China) (5.6). Exome capture was performed on the Illumina® platform using KAPA Library Preparation Kit (Illumina, San Diego, CA). Three family members (II5, III8, and III9) had genetic test for *COL4A1* mutation.

### Pedigree Analysis

The index case had a significant family history of stroke. Detailed family history was obtained from the index case and her cousins. There were 18 family members in the genealogical tree of 3 generations. Nine surviving family members were contacted. Eight of them had MRI brain and four had genetic studies. A pedigree of 3 generations was constructed.

### Skin Biopsy

The involvement of blood vessels within the dermis makes skin biopsy a useful adjunct in the diagnosis of PADMAL and other hereditary cSVD [13, 18]. Skin biopsy was performed on the index case (III10) at the age of 43. A small piece of normal-appearing skin tissue was obtained from right lateral femoral skin under local anesthesia with 2% lidocaine. The sample was fixed with 3% glutaraldehyde and impregnated with epoxy resin. Ultra-thin sections were stained with uranium dioxide acetate and lead citrate. The biopsy slides were examined under Phili EM208S transmission electron microscopy.

## Results

### The Index Case

**Fig. 1:**
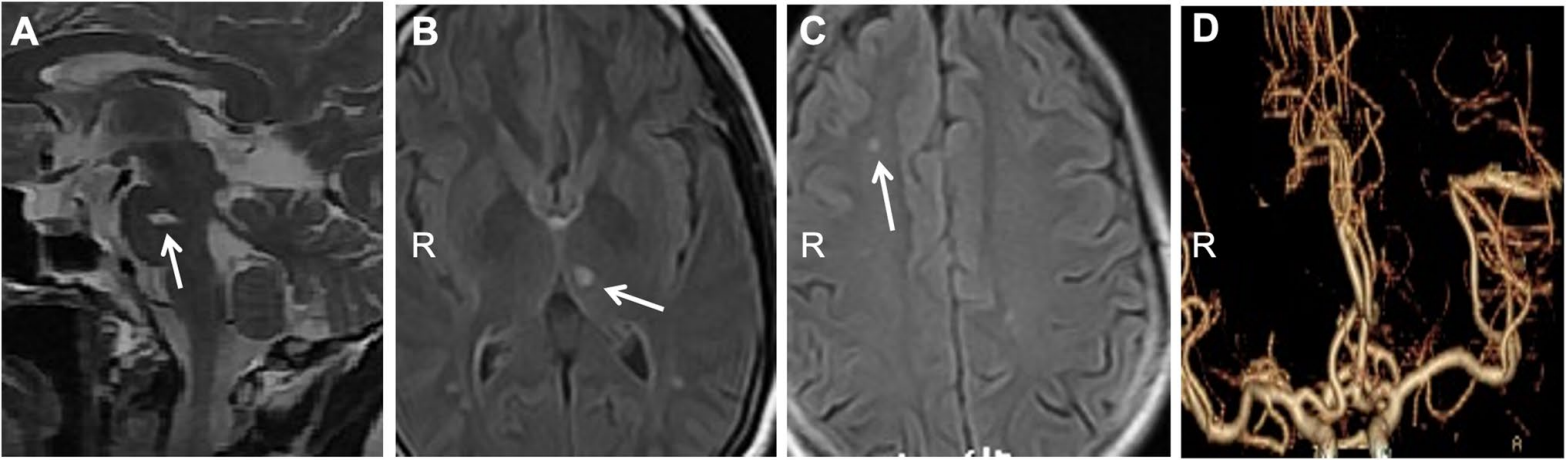
Initial MRI of the index case (III10). MRI brain for minor trauma workup showed abnormal hyperintensity signals in the pons (**a**, T2-weighted), the left thalamus (**b**, FLAIR) and subcortical white matter of the right frontal lobe (**c**, FLAIR). MR angiography of the brain (**d**) was normal

The index case had no significant past medical history. At initial presentation in 2014, her neurological examination was intact. MRI brain for a minor trauma workup showed chronic lacunar infarcts in the pons, left thalamus, and right centrum semiovale (Fig. 1a–c). Brain magnetic resonance angiography (Fig. 1d) and spinal MRI were normal. Transesophageal echocardiography suggested a patent foramen ovale (PFO) and a possible left atrial thrombus, which was later found to be motion artifact. There was no evidence of heart failure, wall motion abnormality or atrial dilatation. No atrial fibrillation or cardiac arrhythmia was detected by continuous dynamic electrocardiogram for 7 consecutive days. Lower extremity Doppler assessment showed no deep venous thrombosis (DVT). She was initially treated with rivaroxaban 20 mg daily for stroke prevention because of PFO and possible left atrial thrombus per American Heart Association/American Stroke Association guidelines [20].

**Fig. 2:**
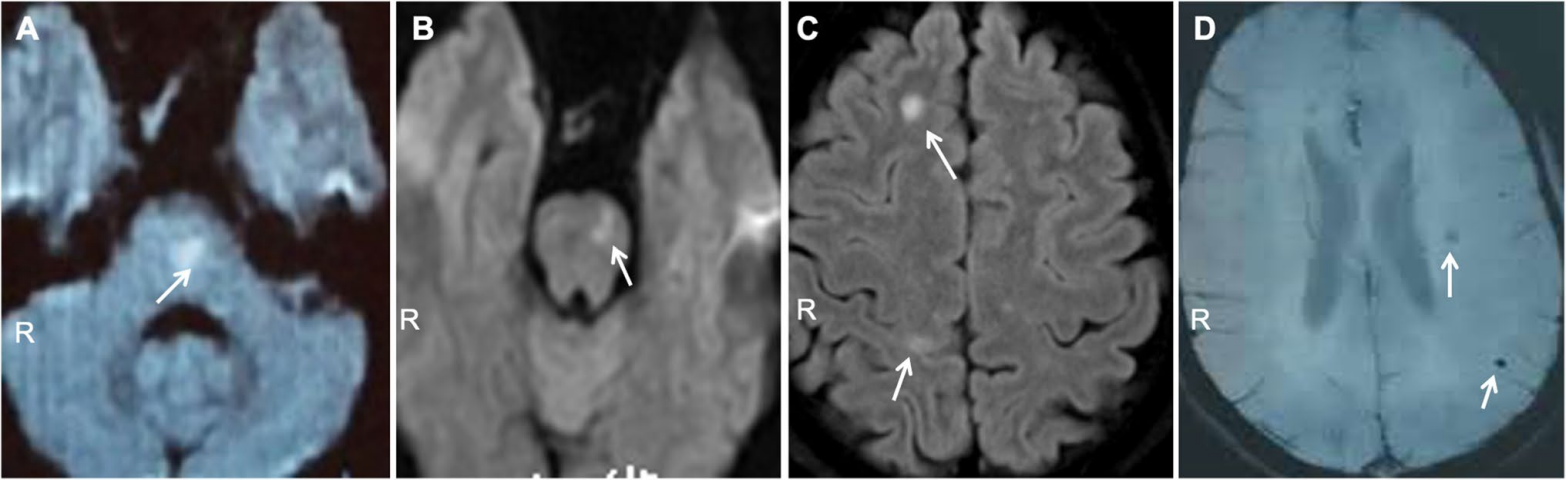
Subsequent MRI brain of the index case (III10). DWI showed an acute infarct in the left paramedian pons (**a** and **b**) and in the subcortical white matter of right frontal lobe and parietal lobe (**c**). Intracerebral microhemorrhages (**d**) were shown on the susceptibility-weighted imaging (SWI)

In 2015, she developed sudden onset of numbness and weakness in the right arm and leg. MRI diffusion weighted imaging (DWI) showed an acute infarct in the left paramedian pons (Fig. 2a). She continued rivaroxaban 20 mg and atorvastatin 40 mg daily for stroke prevention.

In 2016, she had recurrent right-sided arm and leg weakness and numbness. MRI brain showed a new infarct in the left pons on DWI (Fig. 2b). She had PFO closure at that time and was discharged home with clopidogrel 75 mg and atorvastatin 20 mg daily for stroke prevention.

In 2019, she presented with sudden onset of left limb numbness. MRI DWI showed acute infarcts in the subcortical white matter of right frontal and parietal lobes (Fig. 2c). Subcortical microhemorrhages were noted on the susceptibility-weighted imaging (SWI) (Fig. 2d). The patient had multiple lacunar infarcts and subcortical microhemorrhage at age 43. The clinical feature was inconsistent with cerebral amyloid angiopathy.

### The Index Case’s Brother

**Fig. 3:**
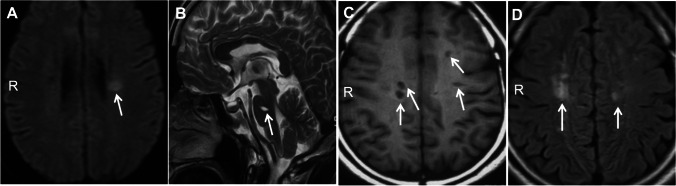
MRI brain of the index case’s brother (III9). MRI showed an acute infarct in the left corona radiata (**a**, DWI) and chronic infarcts in the pons (**b**, T2-weighted) and bilateral subcortical white matter (**c**, T1-weighted and **d**, FLAIR)

The index case’s brother also had early-onset ischemic stroke. He initially developed right limb weakness at age 39 in 2013. MRI showed an acute infarct in the left corona radiata (Fig. 3a) and chronic infarcts in the pons and bilateral subcortical white matter (Fig. 3b–d). Aspirin 100 mg daily and atorvastatin 20 mg daily were started for secondary stroke prevention. He had 5 recurrent strokes despite antiplatelet therapy for secondary stroke prevention. No dual antiplatelet therapy was tried in the index case or her brother.

### Genetic Analysis

**Fig. 4:**
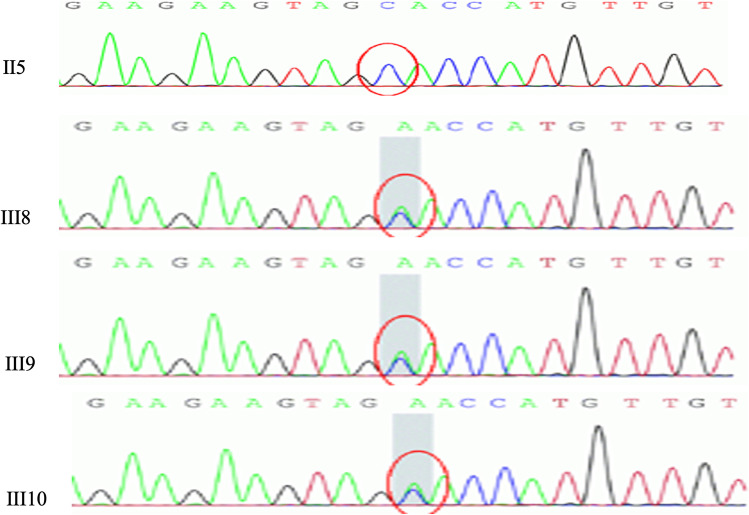
Gene sequencing *COL4A1* gene. The subject III8–10 were shown to have c.*34G > T mutation in *COL4A*1 (marked by the red circle)

**Fig. 5:**
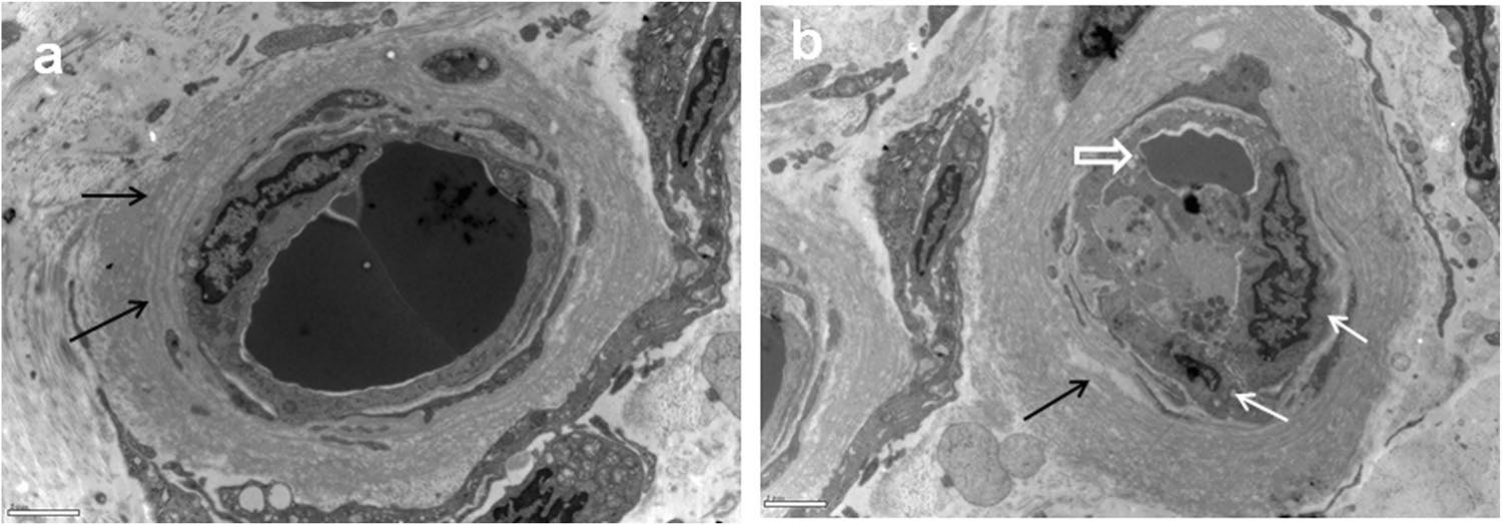
Skin biopsy of the index case. Electron microscope images showed that the vascular basement membrane is thickened and stratified (**a** and **b**, black arrow). Vascular endothelial cell proliferation is shown (**b**, white arrow). The lumen of the vessel becomes narrow (**b**, white hollow arrow). Reginal magnification × 8000; bar 2 μm

**Fig. 6:**
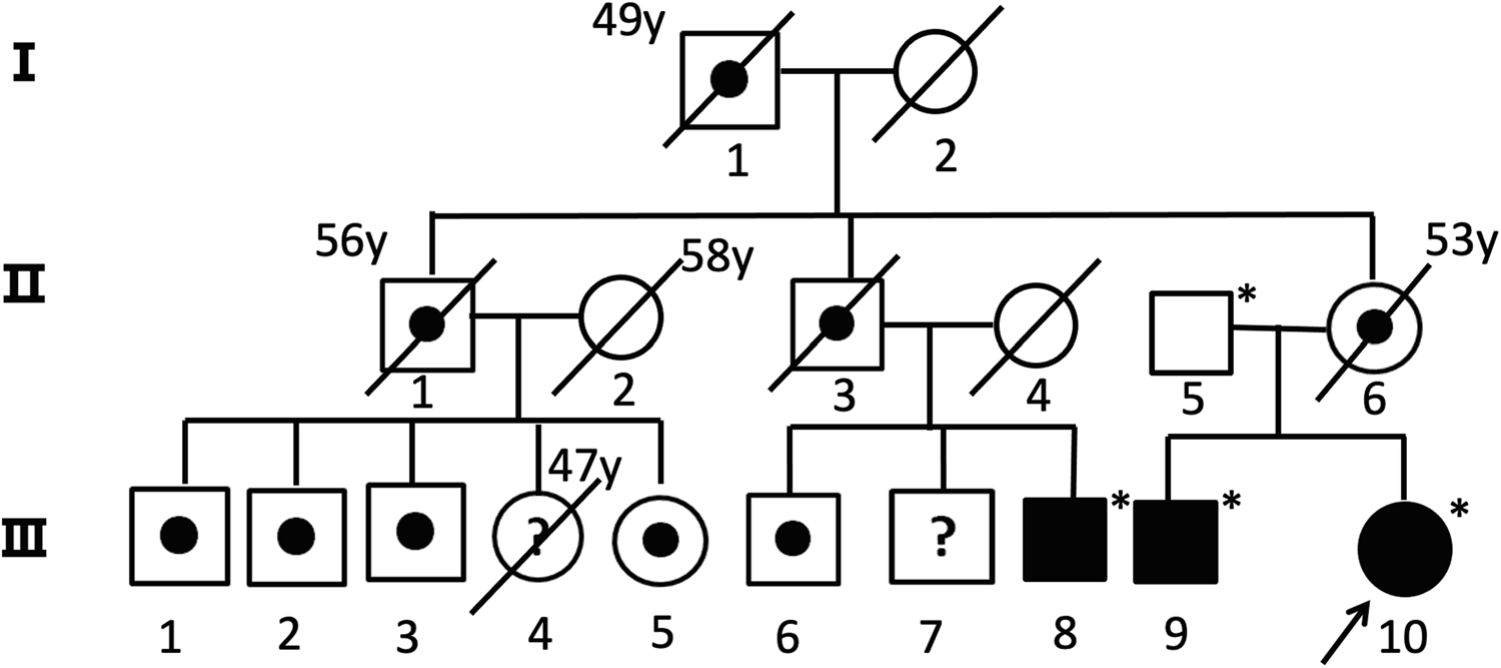
The pedigree of 3 generations. The diagonal line indicates deceased individuals and the known age at death is labeled. The black circle or square indicates individuals with clinically, MRI, and genetically proven PADMAL. *Symbol indicates individuals with genetic test. Black dots indicate individuals with clinically and CT/MRI proven PADMAL. The white circle or square represents healthy individuals. Question marks indicate unknown status. The proband is indicated by the black arrow

Gene testing for *NOTCH3* and *HTRA1* was negative. A novel c.*34G > T variant was detected in the 3′ untranslated region of *COL4A1* gene (chr13-110,802,676) by whole-exome sequencing in the index case (III10 in Figs. 4 and 5). The same variant was also found in index case’s brother and a cousin. The index case’s father (II5) did not carry this mutation (Figs. 4 and 6).

### Skin Biopsy

Ultra-thin section electron microscopy showed thickening and stratification of vascular basement membrane in the dermal layer of the skin, proliferation of vascular endothelial cells, and stenosis of vascular lumen (Fig. 5). These findings support the diagnosis of PAMDAL [13, 18]

### The Pedigree Analysis

Of the 18 individuals in the pedigree of 3 generations, 12 had clinical and CT/MRI evidence of PADMAL (Fig. 6) and no other vascular risk factors (I1, II1, II3, II6, III1–3, III5–6, III8–10). One woman (III4) had history of hypertension and died from cerebral hemorrhage at age 47. It was unclear if she had PADMAL. The diseased individuals all died young, at age 58 or less.

## Discussion

In this report, we demonstrate a novel c.*34G > T mutation in the 3′ untranslated region of *COL4A1* gene in a Chinese family with cSVD. Although the new variant is different from the originally reported mutations (c.*31G > T, c.*32G > T, and c.*35C > A) in patients with PADMAL [13] and the c.*32G > A mutation in patients with multi-infarct dementia of Swedish type [14], the clinical features and skin biopsy results support the diagnosis of PADMAL [13–19].

The onset age of the reported PADMAL patients was 12 to 50 years old [13–19]. It was 38 to 52 in our study. The index case had recurrent lacunar infarcts since age 38. MRI findings were consistent with cSVD rather than cardio-embolism [21]. Extensive workup showed a PFO. Seven-day dynamic electrocardiogram revealed no atrial fibrillation or cardiac arrhythmia. Prolonged cardiac monitoring was not indicated given young age and absence of embolic stroke and atrial dilatation. PFO is present in approximately 25% of the general population [22]. The patient had no embolic stroke or DVT. The PFO was probably an incidental finding. Due to PFO and possible left atrial thrombus, she was initially anticoagulated with rivaroxaban for secondary stroke prevention [20]. The left atrial thrombus was subsequently determined to be a motion artifact. She underwent PFO closure empirically and was transited to antiplatelet therapy after recurrent pontine infarctions on anticoagulation. Despite anticoagulation, PFO closure, and antiplatelet therapy, she had recurrent lacunar infarcts and microhemorrhages. Her brother also had 5 recurrent strokes while on antiplatelet and statin therapy. At that time, there was no evidence to support dual antiplatelet therapy [20]. These results suggest that conventional therapy is not effective for stroke prevention in patients with PAMDAL.

Microbleeds or hemorrhagic stroke were reported in a few patients with PADMAL [13, 19]. They were observed in two subjects (III8 and III10) in our PADMAL pedigree. The index case had recurrent lacunar infarctions and a few subcortical microhemorrhages at age 43. She had no history of hypertension or evidence of cortical hemorrhage. The etiology of the cerebral hemorrhage was likely PADMAL rather than hypertension or cerebral amyloid angiopathy. A female sibling (III4) with a history of hypertension developed cerebral hemorrhage at age 45 and died at age 47. She had no genetic test. The etiology of her hemorrhage was unclear.

The mechanisms of lacunar infarcts and microhemorrhages in patients with PADMAL are likely the overexpression of *COLT4A1* in the basement membrane and frugality of the vessel walls [7, 13]. Therefore, antiplatelet therapy, anticoagulation, and PFO closure are not effective for secondary stroke prevention but potentially harmful. Currently, no specific treatment is recommended for patients with PADMAL [7]. Excessive or prolonged exercise and sporting activities with a high risk of head trauma should be avoided.

In summary, we report a novel c*34G > T mutation in the 3′ untranslated region of *COL4A1* gene in a Chinese PADMAL pedigree. There appears to be a growing spectrum of *COL4A1* overexpression-related cSVD. Patients with subcortical ischemic or hemorrhagic stroke and early-onset dementia are warranted to have genetic study for *COL4A1* gene mutation.

## Data Availability

The data that support the findings of this study are available from the corresponding author upon reasonable request.
